# The Synovial Sealant Variant for Minced Cartilage Repair: A Technical Note and Retrospective Study

**DOI:** 10.1007/s43465-024-01174-8

**Published:** 2024-05-24

**Authors:** Philipp Ahrens, Stefan Hinterwimmer, Matthias Tasser, Lorenz Fritsch, Sebastian Siebenlist, Julian Fürmetz, Julius Watrinet

**Affiliations:** 1 Department of Sports Orthopaedics, Technical University of Munich, Ismaninger Str. 22, 81675 Munich, Germany; 2 OrthoPlus, Lenbachplatz 2a, 80333 Munich, Germany; 3 BG Trauma Center Murnau, Prof. Küntscherstr. 8, 82418 Murnau, Germany

**Keywords:** Cartilage, Defects reconstruction, Biology, One-stage surgery, Autologous

## Abstract

**Purpose:**

Cartilage defects are a common pathology in active people and affect quality of life. A common treatment option is treatment with minced cartilage (MC). As conservative therapy has a limited effect, surgical treatments vary in terms of procedure and results. A modified technique for autologous cartilage repair is presented here.

**Method:**

MC was modified by adding a synovial sealant. This improves the stability of the graft, allowing the cartilage to proliferate. The synovial tissue has the potential to stimulate the implanted cartilage, which promotes healing and regeneration. The clinical and functional results of the modified technique were examined in a retrospective case series.

**Results:**

The technique has proven to be reproducible for retropatellar cartilage defects and is both efficient and cost effective. MC with synovial sealing was performed in ten patients with retropatellar cartilage damage. In the conducted cases serious, 10 patients were available for follow-up after 18 ± 3 months. Patients showed good clinical results in terms of pain (VAS = 1.9, KOOS Pain = 89.7), symptoms (KOOS Symptoms = 83.6), and daily activity (KOOS Activity = 96.6).

**Conclusion:**

The procedure combines the advantages of autologous cartilage repair with a one-stage surgical approach. It utilizes the regenerative potential of synovial tissue while providing improved mechanical stability. This technique offers a cost-effective, autologous solution for full-thickness cartilage defects, and shows promising clinical results in the medium term.

## Introduction

Injuries of the joint cartilage and their repair have been a demanding issue and interesting field of science and surgical developments over decades [1–5]. Since physical work and sporting activity are a matter of health awareness, activity related cartilage defects have a high prevalence in nowadays society [6, 7]. These developments come along with increased magnetic resonance facilities, well-trained physicians, and demanding patients. The easy access to high-resolution magnetic resonance imaging bears plenty of cartilage defects of all sizes and destruction grades [8]. Besides these instrument-based diagnostic findings, pain and defect size are not always related [9]. Knee cartilage injuries are most commonly detected, and in lesions greater than 1.5 square cm, the implantation of newly cultivated and matrix associated cells is reliable but demands two surgical interventions (harvest and implantation) [10–13]. Besides this drawback, the matrix is of bovine origin [14, 15], and costs and regulations are profound. Nevertheless, minced cartilage seems to be a good alternative [16] with successful results dating back to 1983 Albrecht and Borsoe [17] and as well our own experience [18, 19]. The awareness of the strong biopotency of minced chondrocyte containing cartilage, which shows their proliferation and excretion of new extracellular matrix, was the successful starting point of different kinds of surgical techniques [20, 21], but the stable fixation was always problematic because the implanted cartilage chips when first seeded and fixated with fibrin represent a fragile construct during joint motion.

Synovial tissue which covers the whole inner joint capsule is a durable, elastic, and most heavily loaded location of mesenchymal stem cells with growth factors tending for chondrogenesis (MSC) [22, 23]. Besides synovial tissue, MSC are also found in adipose tissue [24–27]. A pathologic benign appearance of synovial-based cartilage producing capabilities can be recognized in the synovial chondromatosis [28–31]. Supported with this background, the here-described surgical procedure has been developed from the necessity of stable particle fixation in counterpart lesions and has matured to our standard procedure for all larger cartilage defects. Additionally, another advantage might emerge from the MSC loading of the sealant tissue. The here-described technique should introduce a manually easy and cost-effective technique for the stable implantation of minced cartilage and its mechanical protection from early shear forces.

## Materials and Methods

### Surgical Technique

**Fig. 1:**
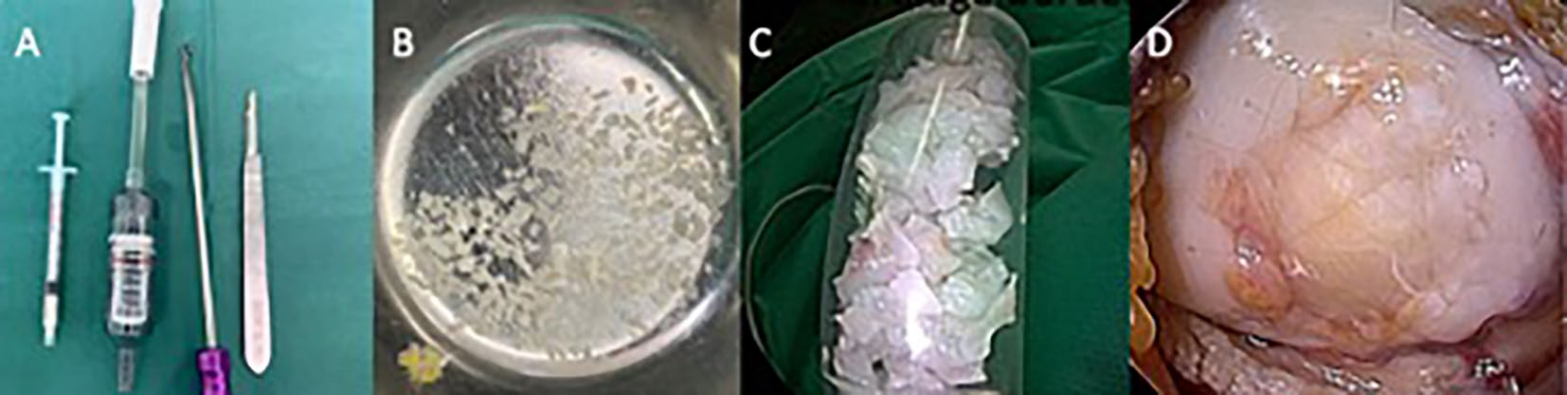
**A** Disposable 2 ml syringe with rubber seal, particle collector GraftNet (Arthrex, Naples), circular abrasor, scalpel for shredding bigger particles for shaver homogenization. **B** Steel basin with irrigation fluid gilled with raw cartilage particles. **C** Processed minced cartilage fragments inside syrringe. **D** Filled defect with stretched synovia and fibirin sealant after particle injection

**Fig. 2:**
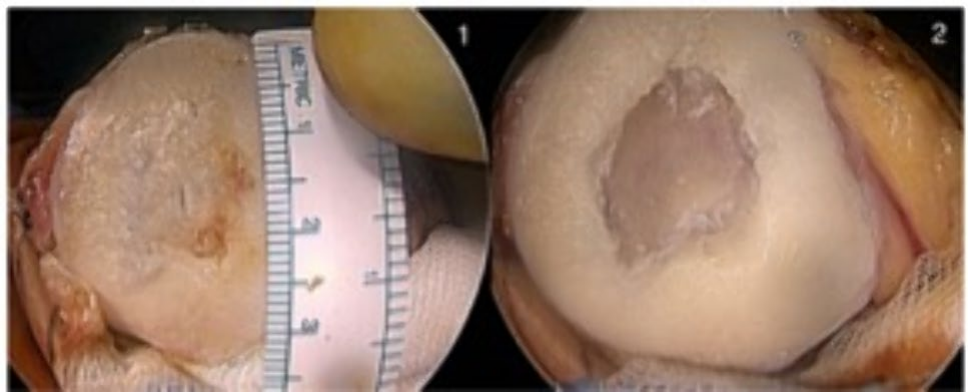
Large degenerative full thinkness cartilage defect of the patella. Using a curette and sharp spoon, the cartilage is harvested and solid defext borders established

**Fig. 3:**
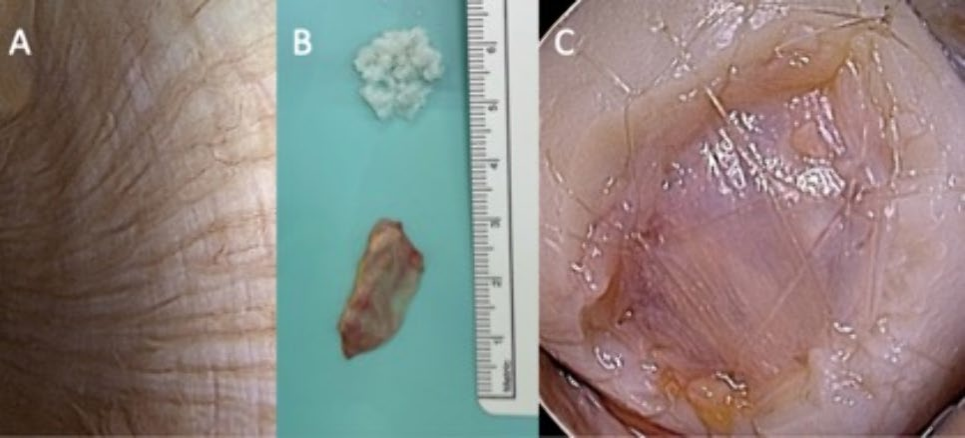
**A** Arthsocopic view on the intraarticular synovial layer. **B** Above, the minced cartilage particles, below harvested synovial flap. **C** Synovial sealant with circumferent stabilizing sutures and pan defect strings to avoid the niveau elevation after cartilage particale injection

The patient position is supine and the use of a tourniquet is recommended to implant the cartilage under bloodless conditions. A perioperative antibiotic prophylaxis is recommended but not without conflicting evidence. In general, the procedure is performed using an open approach which provides better visualization as well as a more exact and faster workflow. After a standard arthrotomy which made the defect area accessible, the harvesting process starts with the collection of cartilage debris using a scalpel and abrasor as seen in Fig. 1A. To provide a sufficient quantity of solid cartilage mass, besides unstable or loose cartilage fragments, it is intended to minimally enlarge the defect by trimming its borders (Fig. 2). The scalpel is used to produce an exact and stable rim wall which provides a solid anchoring of the filament used to fixate the synovial flap. The bed is prepared in full depth using a sharp abrasor [32]. The collected cartilage fragments are stored in a steel bowl with arthroscopic irrigation fluid. Once collected, the cartilage is processed using a Sabre shaver blade (4.0- mm Arthrex, Naples, Florida) and an in-flow collector (e.g., Arthrex, GraftNet, Naples, Florida) which captures the fragments after the shaver passage. To facilitate a moderate paste like mixture, two shaver passages are necessary. The preparation and mincing procedure follows the technique described by Roth et al. [33]; repeated shaver passages make the fragment mix more smooth and enable a better application through the syringe. Additionally, we gather a small amount of adipose tissue, a sufficient portion can be found in the supratrochlear pouch in front of the trochlea grove or below the synovial flap harvesting site. This tissue is also passed through the shaver to release intracellular bioactive growth factors, the material is mixed with the cartilage paste produced [34–36]. Besides the procedure of fragment processing, a synovial membrane is harvested from the most inner side of the joint capsule Fig. 3. The thin synovial layer (Membrana synovialis) is elevated with fine forceps and precisely separated from its underlying fatty tissue. The extracted synovial flap is cleaned off remnants and trimmed to perfectly fit the defect in size and shape. The membrane then is placed on the cleaned defect area and fixed to the cartilage rim wall with multiple filament sutures some over spanning the membrane leveling the cartilage niveau (Glycolon 6–0, Resorba, Germany) Fig. 3C. To inject the cartilage fragments below the membrane, the synovial flap is elevated between two fixation points using an Adson forceps and the cartilage paste is delivered below the synovial tissue using a 2 mm disposable syringe (B. Braun, Melsungen, Germany). The use of a syringe with a centered nozzle and rubber seal turned out to be the most effective Fig. 1A**/C/D**. An adequate filling is realized when the membrane gets slightly stretched and when the filled defect has an equal level to the surrounding cartilage margins. By manipulating the membrane with the fingertip or with the backside of a small forceps, the underlying fragment distribution can be balanced such that all areas of the defect show a solid load of cartilage particles. The defect area is sealed with fibrin (Tissel, Baxter, Oberschleißheim, Germany) to add more stability. The sealant procedure is finished when the clot shows a slight milky appearance. The patella is returned to its physiological position and the joint capsule is closed with standard sutures using Vicryl.

## Case Series

To evaluate the clinical outcome, patients in this case series were followed up retrospectively between January 2021 and November 2021 for at least 12 months after surgery. Inclusion criteria were isolated focal retropatellar cartilage damage without concomitant injuries. Objective and subjective clinical outcomes were determined using the KOOS Score, Tegner Score and VAS.

The study was conducted in accordance with the Declaration of Helsinki, and approved by the Ethics Committee of the Bavarian Medical Association (Nr. 2022–1195). Informed consent was obtained from all subjects involved in the study.

## Results

### Midterm Clinical Outcome: A Case Series

Ten patients with retropatellar cartilage damage ICRS grade four were treated with the presented technique. The group consisted of four women and six men with a mean age of 35 ± 15 years (range 17–56 years) and BMI of 24 ± 4 kg/m^2^ (Table 1). The follow-up period was 18 ± 3 months. After operation, each patient underwent a structured rehabilitation regimen (Table 2). The mean range of motion (ROM)  three to six months postoperatively was 2.3–0-125.7 as measured using the neutral-zero method.

**Table 1:** Summary of demographic characteristics

Demographic data	
Male sex	60% ( *n* = 6)
Age [years]	35 ± 15 [range 17 – 56]
BMI ($\frac{kg}{{m}^{2}})$	24 ± 4
Follow Up [months]	18 ± 3

**Table 2:** Postoperative rehabilitation regimen. *ROM* range of motion

Rehabilitation regimen	
	ROM Knee Extension / Flexion
1. – 2. Week	0° / 0° / 30°
	Additional partial weightbearing (20 kg)
3. – 4. Week	0° / 0° / 60°
4. – 6. Week	0° / 0° / 90°

Eight of the 10 patients had no pain at rest at the follow-up time point. The mean pain level in motion was 1.9 ± 1.6 according to VAS. The sports activity level in the Tegner score was high after treatment with a median value of 5 (range 3–9). The KOOS score showed good results in the domains “pain” (89.7 ± 6.0), "symptoms" (83.6 ± 15.4), and “activity and daily life” (96.6 ± 3.5) (Table 3). In the domains “functionality in sports and leisure activities” (69.1 ± 23.1) and “quality of life” (57.4 ± 22.0), patients reported limitations with large individual variations.

**Table 3:** Functional outcome after synovial sealant variant for minced cartilage

Functional outcome	
VAS	1.9 ± 1.6
Tegner Score	5 [range 3 – 9]
KOOS	
Pain	89.7 ± 6.0
Symptoms	83.6 ± 15.4
Activity and daily life	96.6 ± 3.5
Functionality in sports and leisure activities	69.1 ± 23.1
Quality of life	57.4 ± 22.0

## Complications

No major complication was observed and revision surgery was not needed in this case series. Two patients experienced joint effusion postoperatively, while two others complained of an initial extension deficit. Treatment was successful with conservative treatment in these cases.

## Discussion

Since minced cartilage as a treatment option was introduced in the 1980s, many different techniques and procedures have been published. There is a wide variety of recommendations and experiences facilitating and processing the cartilage pieces, but until now, there is no consensus which technique is to be favored. Nevertheless, there are different cartilage cultivation and implementing approaches available on the marked, but all with the disadvantage of 2-stage interventions, one for harvesting the cartilage and one for implantation of the cultured material. In the most popular techniques, xenograft membranes are used where the cultured cells are imbedded; this procedure works without major complications as far as we know but it is still relaying on a xenograft. Differing from these techniques, we use an autologous synovial tissue flap from the inner side of the joint capsule which seems to be a very good harvesting site. We started using it at first only for opposite defects (Trochlea / Patella) but later changed our procedure that the covering of all defects is now our standard procedure. The thin synovial layer covers the repaired defect physically and leads to more mechanical stability for the first weeks, besides that synovia is a well-recognized source of human mesenchymal stem cells and biopotent co-factors [22, 37–40]. From our view, these attributes might enable a better milieu for the cartilage seeding, ingrowth, and maturing process [41].

Cartilage damage and degeneration is always accompanied by inflammatory processes sidelined by biochemical pathways and transmitter-related disturbances [22, 42]. With respect to this knowledge, it is mandatory even to encounter this course. Recent studies suggest that adipose tissue has the potential to be a strategic partner in joint preservation and might help in down slowing arthritic recession while supporting joint reparation [34, 36, 43–46]. Known for its role in inflammatory modulation especially in arthritic joints [42, 45], again also adipose tissue is a reliable source of stem cells with unique chondrogenic potency [36, 43, 44, 47]. Since efficiency and the reduction of surgical interventions are important for the patient, one-stage approaches are our personal favorite. But there are more rational factors influencing our processing. Supporting our attempt recently literature reported that mincing devices do not significantly affect chondrocyte viability, whereas further outgrowth and proliferation are not influenced [48]. As far as we know, sharp instruments provide the best quality of cartilage fragments, this can be easily processed using the technique as described by Salzman et al. 2020 [49]. Observing the literature published, the results appear to be promising and the knowledge of cell differentiation, adaption, and organization to their demands has been increased [50]. Implanted cells once placed into their natural habitat start to rearrange, adhere, grow, proliferate and maturate in regard to their requirements [49, 51, 52]. Massen et al. [53] reported for the use of a second-generation MCI lowered pain and enhanced knee function scores 2 years after surgery. But direct comparison studies are outstanding at this time.

The technique described here is favorable related to socioeconomic [54], socioecological as well as biologic issues, and represents a useful approach including all facets of actual knowledge in regard to mesenchymal stem cells, fat cell-derived factors, cartilage proliferation, cartilage facilitation, and implantation processes. The presented technique is a single-stage surgery and allows to treat full-thickness cartilage defects. Until now, there are no costs or regulations which hinder the technique as it has been seen in others [55].

**Fig.4:**
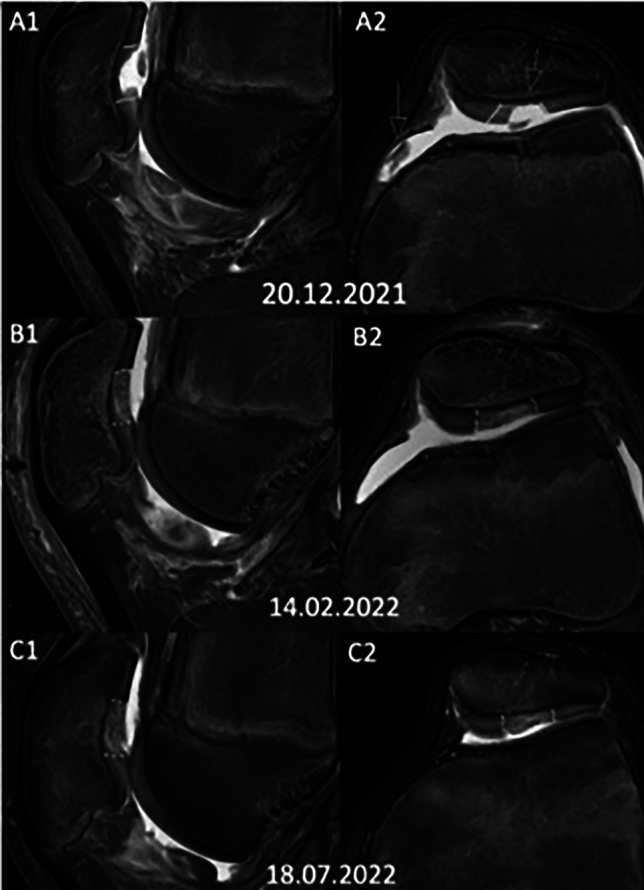
**A1/A2** MRI scan of traumatic full-thickness cartilage defect at the central patella (arrows, defect zone yellow lines). **B1/B2** MRI scan 6 weeks after surgery showing comminuted cartilage and synovial sealant. C1/2 MRI scan 6 months after the procedure. After complete rehabilitation, the patient is pain free and has already resumed his ice hockey career

Besides this subject, one important disadvantage is the limitation of donor side cartilage areas. Multiple injuries or larger defects might overburden the harvesting side that other techniques seem to be more favorable. In some rare cases, the harvesting of additional cartilage fragments has been required by exploring another area, in this situation the deepest margin of the trochlea, where the margin transits into the intercondylar tunnel is another appropriate area without mechanical joint motion. A well-trained and experienced team fastens the application and allows to reduce inappropriate contact times which reduces all common risks of surgery. In our setting, one person is assigned to clear the synovial flap from remnants as well as for shaver homogenization of the cartilage fragments. Using the described technique and instruments, the surgical intervention lasts 40–60 min which supports the healing response and post-surgical rehabilitation. We use of a tourniquet, nevertheless, we acknowledge that there is no evidence in regard to arthroscopic visibility, post-surgical pain killer consumption, and effects for recovery [56]. In many cases, the procedure is accompanied by different other interventions; leg axis correction, ACL, or MPFL reconstruction are the most common co-treatments we perform alongside cartilage repair. The feasibility and biological potency of the procedure has been found in vitro as well as in vivo studies. At this stage, we support any kind of clinical studies. All patients were seen regularly after 6 weeks, as well as 3, 6, 12 month. Until now, all patients report of a meaning full reduction of pain while some also report an ongoing pain full crepitus which might be explainable by the change of the cartilage surface after the procedure. Also, magnetic resonance imaging in our standard 6-month post-surgery underlines the overall satisfaction of the patients by promising radiological results (Fig. 4).

## Conclusion

**Fig. 5:**
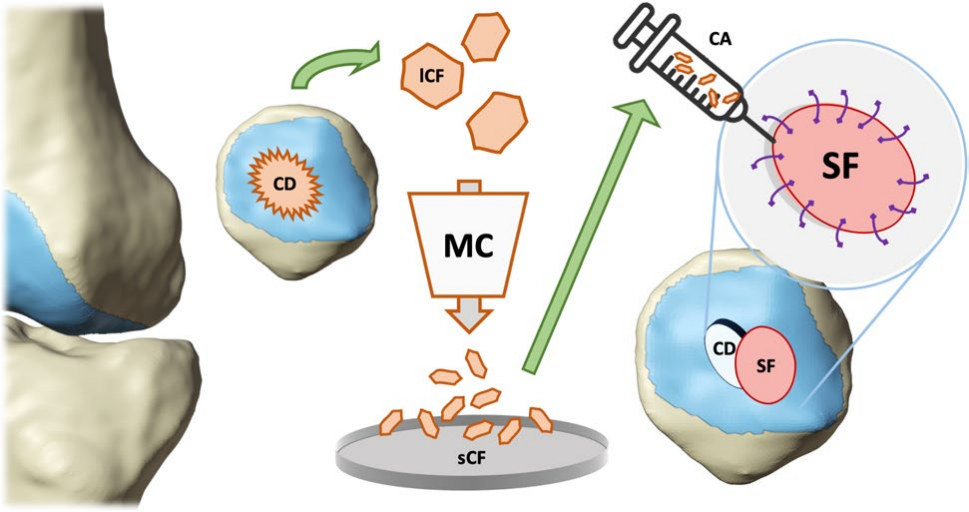
The graphical abstract illustrates a the synovial sealant variant for minced cartilage repair. Initially, a retropatellar chondral defect (CD) is identified in the patellar region. Subsequently, cartilage debridement is performed. Minced cartilage (MC) is then prepared. A autologus synovial flap (SF) is sutured over the defect, and minced cartilage (sCF) is injected (CA) underneath the secured flap to promote cartilage regeneration *CD* retropatellar chondral defect, *lCF* large cartilage fragment, *sCF* small cartilage defect, *MC* minced cartilage, *CA* cartilage application, *SF* synovial flap

The procedure combines the advantages of autologous cartilage repair with a one-stage surgical approach. It utilizes the regenerative potential of synovial tissue while providing improved mechanical stability. This technique offers a cost-effective, autologous solution for full-thickness cartilage defects showing promising clinical results in the medium term. (Fig. 5)

## Data Availability

The patient datasets analyzed in this study is not publicly available.
